# Evaluation of Hypoglycaemia with Non-Invasive Sensors in People with Type 1 Diabetes and Impaired Awareness of Hypoglycaemia

**DOI:** 10.1038/s41598-018-33189-1

**Published:** 2018-10-03

**Authors:** Ole Elvebakk, Christian Tronstad, Kåre I. Birkeland, Trond G. Jenssen, Marit R. Bjørgaas, Kathrine F. Frøslie, Kristin Godang, Håvard Kalvøy, Ørjan G. Martinsen, Hanne L. Gulseth

**Affiliations:** 1 Department of Clinical and Biomedical Engineering, Oslo University Hospital, Oslo, Norway; 2 Department of Endocrinology, Morbid Obesity and Preventive Medicine, Oslo University Hospital, Oslo, Norway; 3 Department of Organ Transplantation, Oslo University Hospital and University of Oslo, Oslo, Norway; 4 Metabolic and Renal Research Group, Faculty of Health Sciences, UiT The Arctic University of Norway, Tromsø, Norway; 5 Department of Endocrinology, St. Olavs Hospital, Trondheim University Hospital, Trondheim, Norway; 6 Department of Clinical and Molecular Medicine, NTNU – Norwegian University of Science and Technology, Trondheim, Norway; 7 Norwegian National Advisory Unit on Women’s Health, Oslo University Hospital, Oslo, Norway; 8 Department of Physics, University of Oslo, Oslo, Norway

## Abstract

People with type 1 diabetes and impaired awareness of hypoglycaemia (IAH) are prone to severe hypoglycaemia. Previous attempts to develop non-invasive hypoglycaemia alarm systems have shown promising results, but it is not known if such alarms can detect severe hypoglycaemia in people with IAH. We aimed to explore whether a combination of non-invasive sensors could reliably evaluate hypoglycaemia (plasma glucose (PG) minimum 2.5 mmol/L) in people with IAH. Twenty participants with type 1 diabetes and IAH underwent randomly ordered, single blinded hyperinsulinemic euglycaemic and hyperinsulinemic hypoglycaemic clamps. Sweating, skin temperature, ECG, counterregulatory hormones and symptoms of hypoglycaemia were assessed. Overall, we were not able to detect clamp-induced hypoglycaemia with sufficient sensitivity and specificity for further clinical use. As a post-hoc analysis, we stratified participants according to their ability to identify hypoglycaemic symptoms during hypoglycaemic clamps. Five out of 20 participants could identify such symptoms. These participants had a significantly higher adrenaline response to hypoglycaemia (p < 0.001) and were reliably identified by sensors. Based on our observations, a non-invasive alarm system based on measurement of sweating responses and ECG changes during hypoglycaemia might provide an alert at a plasma glucose concentration around 2.5 mmol/L if an adequate sympatho-adrenal reaction is elicited.

## Introduction

Severe hypoglycaemia is one of the most feared complications among people with type 1 diabetes^1^. People with impaired awareness of hypoglycaemia (IAH)^2^ have reduced ability to recognize and respond to low plasma glucose (PG), with subsequent risk of confusion or unconsciousness^3^. IAH afflicts 17–28% of people with type 1 diabetes when assessing hypoglycaemia awareness status using the Clarke and Gold questionnaires, and it increases the risk of severe hypoglycaemia by sixfold^4–8^. In a pilot study, a system to monitor the autonomic responses to variations in PG in people with type 1 diabetes with normal awareness of hypoglycaemia has shown promising results^9^. To our knowledge, no such alarm system has been tested in people with IAH. During hyperinsulinaemic hypoglycaemic clamp studies, patients with IAH have initial sympathoadrenal activation at lower PG-levels than people with normal awareness^10^. It is not known whether this alters the response threshold of sensors detecting the autonomic signals. Our aim was to study the physiological responses during hypoglycaemia in people with IAH by symptom scoring, hormonal responses and non-invasive sensors, and explore whether hypoglycaemia-induced autonomic responses that do not induce symptoms can be measured non-invasively in people with IAH. Study participants with type 1 diabetes and IAH underwent euglycaemic and graded hypoglycaemic hyperinsulinaemic clamps to assess if any combination of non-invasive sensor measurements (four sweat sites, heart rate (HR), corrected QT-time (QTc) and skin temperature) could detect hypoglycaemia with nadir around 2.5 mmol/L.

## Results

In total, 21 participants were recruited, 20 completed both hyperinsulinemic hypoglycaemic (HYPO) and hyperinsulinemic euglycaemic (EU) clamps. One participant was excluded due to failure to maintain intravenous lines during the first clamp.

### Subject characteristics, nadir plasma glucose and insulin sensitivity

Subject characteristics are presented in Table 1. Mean age was 41 (standard deviation (SD) 11) years, body mass index (BMI) 25.1 (2.8) kg/m^2^, HbA_1_c 6.4 (0.8) % (46.7 (8.4) mmol/mol) and diabetes duration 23 (14) years. Mean nadir PG during hypoglycaemic clamps was 2.3 (0.2) mmol/L, and mean insulin sensitivity measured from EU–clamps as glucose infusion rate (GIR) was 5.7 (2.0) mg/kg/min (Table 2). All participants had normal kidney function (estimated glomerular filtration rate (eGFR) within normal limits), and none had a history of retinopathy, autonomic neuropathy or peripheral sensori-motor neuropathy. None of the participants had any recordings of PG < 3 mmol/L during the 48 h before the clamps, but three had PG < 4 mmol/L (3.6, 3.6 and 3.7 mmol/L) the night before the EU-clamp and three had PG < 4 mmol/L (3.4, 3.4 and 3.8 mmol/L) the night before the HYPO-clamp.

**Table 1:** Subject characteristics (mean (SD)).

	All participants (n = 20)	Reaction group (n = 5)	Non-reaction group (n = 15)	p-value, difference between Reaction and Non-reaction group
Age, years	41.1 (10.9)	30.6 (11.1)	41.9 (11.1)	0.57
Diabetes duration, years	23.0 (13.8)	17.4 (10.3)	24.9 (14.6)	0.31
Sex, M/F	8/12	1/4	7/8	0.60
Height, cm	170.2 (9.1)	167.3 (8.1)	171.2 (9.5)	0.42
Weight, kg	73.1 (12.1)	71.6 (16.0)	73.6 (11.2)	0.75
BMI, kg/m^2^	25.1 (2.8)	25.3 (3.6)	25.1 (2.7)	0.86
HbA1c, %	6.4 (0.8)	7.0 (0.9)	6.2 (0.7)	0.07
HbA1c, mmol/mol	46.7 (8.4)	52.8 (10.0)	44.7 (7.1)	
Insulin, IU/kg/day	0.64 (0.19)	0.56 (0.1)	0.67 (0.21)	0.29
Gold score^a^	5.1 (1.0)	4.8 (1.3)	5.2 (0.9)	0.61
Clarke score^a^	5.0 (1.1)	5.2 (1.1)	4.9 (1.2)	0.67

^a^A score of ≥4 implies IAH.

**Table 2:** Nadir plasma glucose during hypoglycaemic clamps, and glucose infusion rates at the end of euglycaemic clamps (mean (SD)).

	All participants (n = 20)	Reaction group (n = 5)	Non-reaction group (n = 15)	p-value, difference between Reaction and Non-reaction group
Nadir PG^a^ (mmol/L)	2.3 (0.2)	2.5 (0.1)	2.3 (0.2)	0.04
GIR^b^ (mg/kg/min)	5.7 (2.0)	4.1 (0.9)	6.2 (2.0)	0.008

^a^PG: Plasma glucose. ^b^GIR: Glucose infusion rate.

In addition to presenting data for the whole group, we did a post-hoc stratification; participants were divided into two groups based on whether they were able to recognize that they had low PG during the HYPO-clamp (“Reaction group”, n = 5) or not (“Non-reaction group”, n = 15) to see if there were differences in their physiological response to hypoglycaemia. Median (or mean where applicable) values of measures for these groups during HYPO-clamp are presented alongside the HYPO-clamp and EU-clamp results for the whole group. Subject characteristics did not differ significantly (significance level p < 0.05) between the Reaction group and Non-reaction group (Table 1).

### Sweating

**Figure 1:**
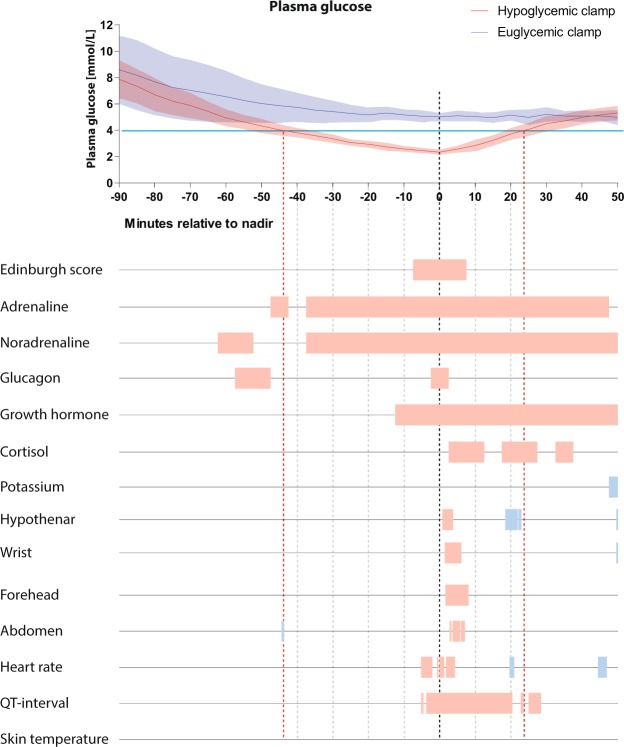
Plasma glucose (PG), mean (whole line) and 95% CI (colored area) for hypoglycaemic clamp (HYPO) (red) and euglycaemic clamp (EU) (blue). All x-axes have nadir PG at zero minutes; other values are minutes relative to zero. Red and blue boxes on the time axis for the different parameters denote significantly (p < 0.05) higher values in the HYPO-clamp (red) or in the EU-clamp (blue). Red dotted vertical lines denote the time frame for the hypoglycaemic part (below 4 mmol/L) of the hypoglycaemic clamp, and black dotted vertical line denotes PG nadir.

**Figure 2:**
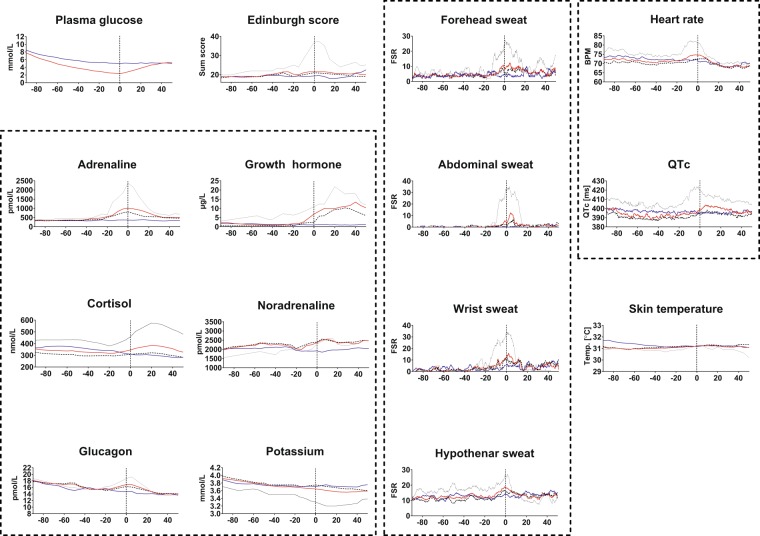
Hormonal and symptomatic responses during HYPO-clamp (red) and EU-clamp (blue). Red and blue lines: Medians (or means, if applicable).Colored areas: 95% CI. Black lines: medians or means for the Reaction group during HYPO-clamps. Dashed black lines: medians or means for the Non-reaction group during HYPO-clamps. Time = 0 is marked with a vertical dashed line. Dashed boxes circumscribe related graphs (blood tests, sweat measurements and ECG-data).

In all four sites, there were significant differences in sweating between the EU- and HYPO-clamp within 5 min. after PG nadir (Fig. 1). The hypothenar site differed from the other sites by a higher baseline sweat response during both EU- and HYPO-clamps, and a less marked response to hypoglycaemia. Average sweating levels were markedly higher in the Reaction vs. Non-reaction group at nadir (p-values: hypothenar 0.04, wrist 0.003, forehead 0.001, abdomen 0.008) (Fig. 2).

### Edinburgh hypoglycaemia symptom scale (EHSS)

The symptom scores increased during the HYPO-clamp, with significant differences between the EU-clamp and HYPO-clamp around glucose nadir (Fig. 1). Six of 20 participants had an increase (≥4, see statistical methods) in symptom scores, and of those, four reported having a hypoglycaemic response during the HYPO-clamp, and one reported having a response that could imply high as well as low PG (these five participants constitute the “Reaction group”). None of the remaining 14 participants were aware that their glucose levels were low during the hypoglycaemic clamp.

Autonomic symptoms were the most commonly reported symptoms in the EHSS^11–13^, (see Table 3).

**Table 3:** Number of participants with increase (≥4) in Edinburgh symptom score.

Edinburgh Symptom Score	All participants (N = 20)	Reaction group (N = 5)	Non-reaction group (N = 15)	p-value, difference between Reaction and Non-reaction group
Overall	6	5	1	<0.001
Autonomic	4	4	0	0.001
Neuroglycopenic	2	2	0	0.05
Non-specific	1	1	0	0.25

### Counterregulatory hormones

Figure 1 displays the significant differences between the EU-clamps and the HYPO-clamps for each measurement. At PG nadir, all hormones except cortisol were elevated compared to the corresponding time in the EU-clamp. In the Reaction group vs. the Non-reaction group, adrenaline (p < 0.001), growth hormone (GH) (p = 0.033) and cortisol (p = 0.012) increased significantly at PG nadir (Fig. 2).

### ECG and skin temperature

Heart rate (HR) during hypoglycaemia was significantly increased around PG nadir (Fig. 1). During two short periods after PG nadir, HR during HYPO-clamp was significantly slower than at the corresponding time during the EU-clamp. During the HYPO-clamp, the mean QTc increased significantly from about 5 minutes before PG nadir to 20 minutes after nadir (Fig. 1), and this QTc-increase was positively correlated with the increase in adrenaline, (p = 0.04, n = 20). During hypoglycaemia, HR and QTc increased in the Reaction group compared to the Non-reaction group (Fig. 2), but this increase was not statistically significant at nadir (HR p = 0.07 and QTc p = 0.05).There were no significant differences in skin temperature between EU- and HYPO-clamps.

### Combining non-invasive sensors

**Figure 3:**
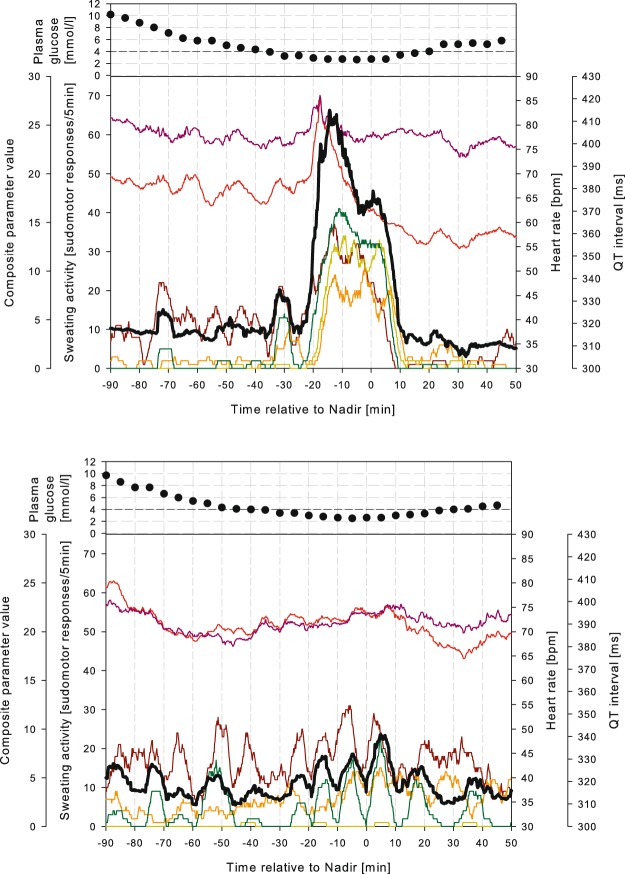
Two examples of sensor recordings during hypoglycaemic trials, showing one case from the Reaction group (upper graph) and one case from the Non-reaction group (lower graph). PG above for comparison. Sweating activity is shown in brown (hypothenar), orange (forehead), yellow (abdomen) and green (wrist) lines. Red line: HR. Purple line: QTc. Black line: combined parameter (unitless).

**Figure 4:**
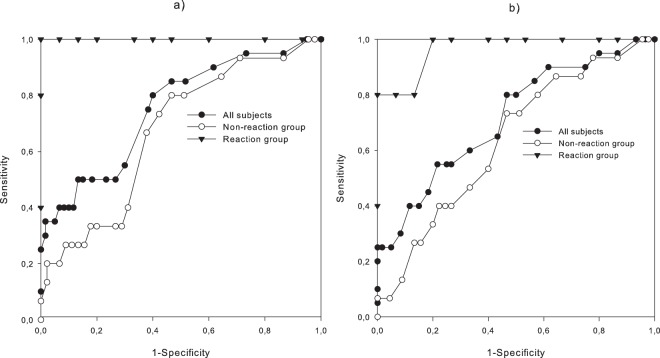
(**a**) ROC-plot for a combined parameter including HR, QTc, wrist sweat and abdominal sweat. (**b**) ROC-plot for a combined parameter of HR and wrist sweat.

We explored several different combinations of measurements for hypoglycaemia detection. We found that the combination of sweating at the wrist and abdomen together with HR and QTc provided optimal detection of hypoglycaemia: Skin temperature, forehead sweating and hypothenar sweating did not improve the performance of the predictor. Figure 3 shows this predictor applied to two participants, one from the Reaction group, and one from the Non-reaction group. We also investigated a more practical predictor based on a sensor combination of only wrist sweating and HR, possible to measure by a wrist-watch type of sensor device. We used receiver operating characteristic (ROC) curves for the two predictors, applied to three groups; all participants, the Reaction group and the Non-reaction group (Fig. 4). In our Reaction group, we observed an area under the curve for the ROC (AUROC) of 1.0 for the first predictor, and 0.94 for the second. Corresponding AUROCs for the Non-reaction group were 0.65 and 0.60, and for all participants 0.75 and 0.71, respectively.

## Discussion

In this study, we have investigated symptoms and hormonal responses to insulin-induced hypoglycaemia in people with IAH and combined these data with data from non-invasive sensors, with the aim to evaluate the potential for detection of hypoglycaemia. For all participants with IAH, no combination of measurements did accurately distinguish between hypoglycaemia and euglycaemia. However, in the participants who had some remaining symptoms of hypoglycaemia (the “Reaction group”), a combination of parameters from sensors could reliably identify hypoglycaemia. The hypoglycaemia symptoms in these participants were predominantly autonomic in origin, and they had higher levels of circulating adrenaline during hypoglycaemia (as compared to the “Non-reaction group”), indicating a more pronounced autonomic response^14^.

We found an increase in HR and QTc during hypoglycaemia (as previously reported^15–18^), and a sweat response in all four sites almost simultaneously. While sweat responses and HR attenuated quite fast, the increase in QTc persisted until 30 minutes after glucose nadir. As to hypothenar sweat and HR, there was an opposite effect (more sweating in the EU- clamp vs. HYPO-clamp) 20 minutes after nadir, indicating a possible increased vagal activity on top of an ongoing sympathetic response, with a possible increased risk of cardiac dysrhythmias^17^.

The strengths of this study include the extensive data collection during the two clamps (PG, counterregulatory hormones, symptom scores, ECG, sweating and temperature) that allowed comparison of the subjective experience of hypoglycaemia and the physiological responses over an extended period of time. The use of single blinding and randomized order of hypoglycaemic and euglycaemic clamp may imply high validity of the collected data.

Sejling *et al*.^19,20^ have previously investigated people with IAH during hypoglycaemic clamp, and compared their responses with people with normal hypoglycaemia awareness. Sweating and ECG-changes were not assessed. To our knowledge, there are no previous reports of ECG- findings during hypoglycaemic clamp in people with IAH, and the present ECG-findings are of particular importance as they may corroborate the risk of cardiac dysrhythmias during hypoglycaemia also in people with IAH^15,17^.

We could not detect a significant change in temperature between EU- and HYPO-clamps or between the Reaction group and Non-reaction group during HYPO-clamps. Sejling *et al*. found that there is a decrement in skin temperature during hypoglycaemia, and that this decrement is less in IAH patients^20^. Circumstances that could affect a participant’s temperature include the hand/arm-warmer, placement of the sensor and the ambient temperature in the room. The warming unit held 37 °C during the whole experiment, and room temperature was not strictly controlled. We aimed at keeping the participant comfortable, i.e. if she/he felt cold they were provided a blanket, or if she/he felt warm, we could open a window in another part of the room. Because this is how people would normally adjust their comfort temperature, it is more similar to a situation in which a sensor system would normally be used. Also, we gathered that this was the most sensible way to exclude any influence from the warming unit, as long as the participant felt that she/he had a comfortable temperature, the warming unit would not change overall body temperature. It also should be noted that the warming unit and the temperature sensor were not attached to the same arm. Since the fall in temperature during hypoglycaemia is likely adrenaline related, the fact that most of the participants did not have a very marked hypoglycaemic reaction (and a poor adrenaline response) may be an explanation to our measurements. However, we did not find a significant difference between the Reaction group and Non-Reaction group either, but as can be seen in Fig. 2 there is a downward trend in the Reaction group from nadir and onwards during HYPO-clamps. A longer clamp procedure or a more pronounced hypoglycaemia might have triggered a stronger response, or larger groups for comparison might have shown significant results.

Three participants experienced low PG the night prior to the HYPO clamp and this may have blunted their autonomic responses during the subsequent clamp. However, this mild hypoglycaemia (3.4 mmol/L at the lowest in two participants) was quickly normalized. Since the acute autonomic response to hypoglycaemia has been reported to occur at lower PG levels in people with IAH than in people with normal awareness^10,21^ and at substantially lower levels than in the present trial, we find it unlikely that a short period with PG ~ 3.5 mmol/L may have influenced the present results.

Our study participants were supine during the clamps, and as previously reported^22^, this may have reduced their ability to detect hypoglycaemic symptoms. But since they were aware that they hypoglycaemia could be induced, they could also have been more attentive to symptoms. Attenuation of hypoglycaemia symptoms by a high insulin level has also been proposed^23^. However, the serum insulin levels in our participants ranged between 461 and 896 pmol/L during clamps, and these levels are less likely to affect symptom detection.

The IAH classification was done using two different methods, the Clarke score^7^ and the Gold score^8^, which have been validated and show good concordance in people with type 1 diabetes^24^. However, five persons with IAH were able to identify the hypoglycaemic clamp, although the symptom scores are remarkable similar between the groups (Table 1). This may reflect the dynamic nature of the IAH syndrome^25^, or it may be interpreted as a weakness of the self-report questionnaires, implying a possibility for misclassification. In that respect, our “Reaction group” may have been misclassified as IAH. Furthermore, avoidance of hypoglycaemia for 48 hours might have improved symptom responses^26^.

It could be argued that PG was not lowered sufficiently to elicit responses in people with IAH. However, as our aim was to explore if we could measure small physiological responses that were not obvious to the patient, eliciting strong sympathetic responses (which would immediately be recognized by the patients) would actually be contrary to our aim. We decided that if we could detect physiological responses at 2.5 mmol/L, there would still be ample time to correct the situation. However, mean nadir PG was actually 2.3 mmol/L in our study, and the Reaction group had higher nadir PG of 2.5 mmol/L (Table 2), which excludes the possibility that the Reaction group had sympathetic activation because of lower PG nadir.

It could be questioned if we should have a group with normal awareness in our study. Ideally, a group with normal awareness should be included if the aim was to look at the differences between groups with or without a hypoglycaemic reaction during hypoglycaemia. This was not our original objective since we did not expect typical hypoglycaemic reactions in participants with IAH at this PG level. Still, when some of them had a reaction, the differences from the Non-reaction group were so obvious that we found it appropriate to include it in our findings. The two groups were not matched, but no significant differences in group characteristics were found, and our results would therefore not be biased by baseline differences.

A hypoglycaemic clamp may be called unphysiological and not a good approximation to a hypoglycaemic episode under normal conditions. We tried to emulate a normal hypoglycaemic episode by choosing to do a clamp with steadily falling PG with a rate of decline that can be observed in CGM monitoring, as opposed to a stepped clamp. But a laboratory setting is naturally quite far from a daily life episode, but to do a clamp procedure with frequent blood sampling there is unfortunately no good alternative. Like all research in the field of non-invasive glucose measurement, the biggest hurdle is real life testing.

There have been previous attempts to develop a non-invasive hypoglycaemia alarm. The Sleep Sentry® is a watch-like hypoglycaemia alarm based on sweat detection that was developed in the 1980’s, and is one of two commercially available alarms today (another device called HSA-01 is available in the UK, and seem to function in the same way). The now retracted HypoMon®^27,28^ was another hypoglycaemia alarm, which used several ECG-features to identify hypoglycaemia in adolescents and young adults. A pilot study from Schechter *et al*.^9^ assessed heart rate, sweating (one site only), skin temperature and tremor to detect spontaneous nocturnal hypoglycaemic episodes in 10 adolescents with type 1 diabetes. Compared to these studies, we have a larger array of measurements to assess sympathetic output. The Sleep Sentry only utilizes a single sweat sensor, the HypoMon® only ECG-features, and Schechter *et al*. used only one sweat site and only HR derived from the ECG. We also assessed counterregulatory hormones and hypoglycaemic symptoms in order to better detect hypoglycaemia and investigate whether our measures could detect hypoglycaemia more effectively than patients themselves.

None of the previously developed alarm systems are in widespread use. While some may find the Sleep Sentry® useful, it has poor sensitivity and specificity^29^. Similarly, the HypoMon® failed because of low sensitivity and specificity under real world conditions. The pilot study by Schechter *et al*.^9^ reported good results (sensitivity 100%, specificity 85.7%) in adolescents with normal hypoglycaemia awareness who woke up when spontaneous hypoglycaemia occurred. We have investigated people with IAH that are more likely to not perceive hypoglycaemia, and probably would not wake up during such episodes. Our combined assessments could not detect all episodes of hypoglycaemia, but we were able to look at differences in sympathetic responses in the participants with hypoglycaemia symptoms (Reaction group) and those who could not self-detect hypoglycaemia (Non-reaction group). The present findings are important when evaluating alarm systems based on a sympathetic response, since IAH may be present in up to one in four of diabetes patients^4–6^. Even though alarm systems such as HypoMon® may show good results when tested in a laboratory setting in people with normal hypoglycaemia awareness, similar alarm systems may fail as commercial products if it is not recognized that performance could vary substantially from one individual to another.

Is it impossible to construct a non-invasive sensor system for hypoglycaemia detection? According to our findings, it seems to be difficult for people with a more persistent IAH. In our Non-reaction group, the combined parameters did not perform well enough for clinical use. However, in our Reaction group, the performance of the sensors was excellent. It should be noted that this does not mean that the sensors are completely unable to detect hypoglycaemia in the Non-reaction group, but they could not detect it at this BG level (2.5 mmol/L). Most probably, all participants would have reactions at lower BG levels, but at 2.5 mmol/L it is likely that corrective action can be effected before the patient is unable to act on it due to neuroglycopenia. From Fig. 3 it is evident that setting a cut-off level for the hypoglycaemia detection combined parameter for all participants is very challenging, in light of the differences between individuals with and without a symptomatic (i.e. sympathetic) response. The Clarke and Gold scores were not different in the Reaction vs. the Non-reaction groups, precluding that such scores could be of use to identify people who might benefit from non-invasive sensors. Therefore, the use of combined measurements to detect hypoglycaemia, as in the present study, is probably most useful for people with type 1 diabetes and normal hypoglycaemia awareness, either as an added security, or in situations where they might have reduced alertness, e.g. during sleep.

Several additional challenges may arise if such sensors are to be used as an everyday hypoglycaemia alarm. Sweating, HR and QTc have physiological fluctuations during the day, e.g. during exercise. A wristband assessing sweating and HR might be the most feasible solution and has nearly as good performance as our combined parameter (AUROC 0.94 vs. 1.0 in the Reaction group).

In conclusion, in the present hypoglycaemic clamp study, several ECG parameters, sweating at four sites, counterregulatory hormones and hypoglycaemic symptoms have been simultaneously assessed in people with type 1 diabetes and IAH. We have demonstrated a difference in responses between participants who did and did not recognize hypoglycaemia. Our findings suggest that a sympathetic response with adequate adrenaline output is necessary for the detection of hypoglycaemia with ECG and sweat measurements, and that the lower PG threshold for autonomic activation in people with IAH also alters the response threshold of sensors detecting the autonomic signals. More advanced modelling along with other non-invasive sensors based on electrical or optical properties of skin to detect trends in PG may improve assessment also in people with IAH, and should be subject to further research^30,31^.

## Methods

### Participants

Twenty participants (12 women) aged 18–60 years with type 1 diabetes and IAH were recruited from the outpatient population of Oslo University Hospital, the Norwegian Diabetics Centre, and the Norwegian Diabetes Association. IAH was assessed using the Clarke^7^ and Gold^8^ questionnaires. In the Clarke questionnaire eight questions characterize the participants’ exposure to episodes of moderate and severe hypoglycaemia and assess the glycaemic threshold for, and symptomatic responses to, hypoglycaemia. The Gold questionnaire asks: “Do you know when your hypos are commencing?” The respondents selected a number on a 7-point Likert scale, with 1 representing “always aware” and 7 representing “never aware”. For both tests, a score of ≥4 implies IAH. To be included in the study, the participant should score ≥3 in both tests and ≥4 in at least one of them.

People with heart, lung or kidney disease, hypertension, previous seizures or any condition that could increase the risk of the procedure or influence the hypoglycaemic responses were excluded from participation (see Supplemental Material, Note 1). All participants attended an information meeting and gave written informed consent prior to any investigational procedure. The study was approved by the south-eastern review board (REC South East) of the Regional committees for medical and health research ethics in Norway, and the study was performed in accordance to this approval (approval reference: REK 2013/813).

### Experimental procedures

Each participant had one EU-clamp and one HYPO-clamp performed in random order, and procedures were separated by at least two weeks. Participants were requested to perform frequent self-monitoring to avoid PG values of <4.0 mmol/L 48 hours prior to each clamp^32–34^, and to abstain from alcohol and strenuous physical exercise. Only rapid-acting insulin was allowed 24 hours before each experiment. Participants were hospitalized the evening preceding the clamp, and fasted from 10 pm. Rapid acting insulin was infused intravenously at a variable rate during the night according to an algorithm targeting fasting PG 7–8 mmol/L. Participants were randomly allocated to EU-clamp or HYPO-clamp as first procedure, and blinded as to which type of clamp that was carried out.

A fixed dose of insulin (NovoRapid, NovoNordisk, Denmark) 1.5 mU/kg/min was infused, and glucose 200 mg/mL was adjusted every 5 minutes to maintain PG around 5.3 mmol/L (EU-clamp) or to lower PG gradually to 2.5 mmol/L (HYPO-clamp). The goal was to keep PG at 2.5 ± 0.2 mmol/L for 15 minutes before more glucose was administered to raise PG to euglycaemia. For ethical reasons regarding the safety of our participants, we did not want to reduce PG further. At predefined time intervals (EU-clamp) or predefined PG levels (HYPO-clamp) arterialized venous blood samples^35^ were obtained for measurement of adrenaline, noradrenaline, glucagon, GH, cortisol and potassium. For additional details see Supplemental Material Note 2. At the same predefined time intervals participants scored hypoglycaemic symptoms on a Likert scale from 1 (‘not present’) to 7 (‘present a great deal’) using an expanded version of the EHSS, which has been translated into Norwegian^4,11,12^. Insulin sensitivity was measured as GIR (mg/kg/min) during the last 30 min of the EU-clamp.

### Non-invasive monitoring of physiological measurements

A basic figure of the setup can be found in online Supplemental Material Fig. 1. Sweating on the palm (hypothenar eminence), on the palmar side of the wrist, forehead and abdomen (level of T9 dermatome 2 cm above and to the right of the umbilicus) were assessed with a Sudologger (Biogauge AS, Stabekk, Norway) measuring skin alternating conductance. These measurements were converted into the frequency of skin conductance responses (FSR) reflecting the activation level of the sympathetic nervous system on the specific dermatome^36^. HR and QT-interval were assessed by continuous recording of 3-lead ECG in the lead II configuration, as a substitute for the mean QT-interval from a 12-lead recording^37^. Bazett’s correction formula was used to standardize QT-interval in relation to HR (QTc)^38^. The ECG was recorded using a SC9000XL ECG monitor (Siemens Medical Systems, Danvers, Ma, USA), digitized at 300 samples per second using a NI USB 6009 data acquisition device (National Instruments, Austin, Tx, USA) and processed in real time by a custom-made LabVIEW program (National Instruments).

Skin temperature was assessed every five minutes with the SC9000XL using a Siemens Drager 5204669 temperature probe taped to the left shoulder, not covered by clothing. The setup was found compliant with Annex VIII and X of the Medical Devices Directive 93/42/EEC by a committee at Oslo University Hospital, and approved by the Norwegian foundation for testing and approving of electrical equipment (NEMKO).

### Analytical methods

Glucose was measured in whole blood every five minutes using an YSI 2300 STAT Plus glucose analyzer (YSI Life Sciences, Ohio, United States), and was converted to PG by multiplying with a conversion factor based on each participant’s hematocrit level. Blood samples for catecholamine analyses were collected in vacutainer tubes treated with ethylene glycol tetraacetic acid and glutathione from Sigma–Aldrich (St Louis, MO, USA) and placed on ice. Plasma was separated by centrifugation (3000 rpm, 15 min, 4 °C) and frozen at −80 °C until assayed. Analyses were done using HPLC (Agilent Technologies, Santa Clara, CA, USA) with a reversed-phase C-18 column (Chromsystems, München, Germany) and electrochemical detection (Antec, Leyden Deacade II SCC, Zoeterwoude, The Netherlands) using a commercial kit from Chromsystems. The intra-and inter-assay coefficient of variation (CV) were 3.9% and 10.8% respectively^39,40^. All samples from an individual participant were measured in one run to minimize the interassay variability. Glucagon was analyzed with radioimmunoassay (RIA), CV 7–10% (GL-32K, Millipore corporation, Billerica, Ma, USA)^3^. Due to a change in analytical methods at the Hormone Laboratory, Oslo, Norway, GH of the first five participants were analyzed using immunofluorometric assays (DELFIA) from Perkin Elmer Life Sciences (Wallac Oy, Turku, Finland), and the remaining 15 were analyzed using a non-competitive immunolumonometric assay, CV 6–7% (Immulite 2000, Siemens Healthcare, Llanberis, UK). Cortisol was analysed using a luminescence immunoassay, CV 7% (Immulite 2000, Siemens Healthcare, Llanberis, UK). Potassium was analyzed using an ion selective electrode principle, CV 1–2% (MODULAR, Roche Diagnostics, Indianapolis, USA).

### Statistical methods

For sufficient power, 20 participants were recruited for the study (for details, see online Supplemental Material, Note 3).

The duration of individual clamps varied. We chose a common time span for both clamps based on the HYPO-clamps, spanning from 90 minutes before and 50 minutes after the individual nadirs, hence aligning all participants so that all individual nadirs occured at time = 0. When EU-clamps were performed after HYPO-clamps, measurements were taken at the same time points. In participants who underwent EU-clamp before HYPO-clamp, blood sampling was not always performed at similar clamp duration, and linear interpolation was used to obtain intermediate values between measurements. Then, individual EU-clamps were aligned with corresponding HYPO-clamps so that the starting time was the same, and time points could be compared directly.

For each of the 13 physiological measurements and the symptom score (EHSS), the 20 curve pairs (one EU and one HYPO) were compared pointwise (using interpolated points where needed) over the time range, using paired t-tests for normally distributed differences and the Wilcoxon Signed Rank test upon violation of the normality assumption. The bootstrap method was used to determine corresponding means or medians with pointwise confidence intervals, using the *bootci* function (using the bias corrected and accelerated percentiles method) in Matlab R2016b.

A significant symptomatic response to hypoglycaemia was defined as a score ≥4 higher than the highest score during euglycaemia, (including the two first scores during HYPO-clamp). This would change the score for one symptom from 1 (no symptom) to 5 (in the upper half of the score), or increase the score for two or more symptoms concurrently. For the difference between groups in number of participants with a significant symptomatic response, the Fisher exact test was used (Table 3).

When comparing the Reaction group and the Non-reaction group, we used time point zero of the HYPO-clamp (nadir) only, and tested for difference in all physiological measurements and symptom score. We used the independent samples t-test under the normality assumption and the Wilcoxon Rank Sum test upon violation of the normality assumption. The same tests were used for subject characteristics.

The associations between QTc and adrenaline were assessed by the Pearson product-moment correlation coefficient.

In order to test the ability to detect hypoglycaemia, a composite parameter for the combination of non-invasive sensor measurements was calculated based on a method we have reported previously^36^. ROC analyses were done for the composite parameter value with a variable detection threshold and the corresponding PG level (above or below 4.0 mmol/L) for different combinations of sensor measurements, comparing their detection ability by AUROC. The recordings were split into three euglycaemic periods, (the two periods before and after hypoglycaemia during HYPO-clamp, and one period for the EU-clamp), and one hypoglycaemic period (PG < 4.0 mmol/L in the HYPO-clamp). Detection criteria were defined as follows: A true positive event was registered if at least one (composite parameter) value above the detection threshold within the hypoglycaemic period. A true negative was registered if all values were below the detection threshold for each of the euglycaemic periods. A false positive was registered if at least one value was above the detection threshold in the EU-clamp, and for at least one unique response (not extending into or coming out of the hypoglycaemic period) above the detection threshold during the euglycaemic periods of the HYPO-clamp. A false negative was registered for no values above the detection threshold during the hypoglycaemic period.

## Electronic supplementary material

Supplemental material

## Data Availability

The datasets generated and analyzed during the current study are available from the corresponding author on reasonable request.
