# Author Correction: Evaluation of Hypoglycaemia with Non-Invasive Sensors in People with Type 1 Diabetes and Impaired Awareness of Hypoglycaemia

**DOI:** 10.1038/s41598-019-42218-6

**Published:** 2019-04-16

**Authors:** Ole Elvebakk, Christian Tronstad, Kåre I. Birkeland, Trond G. Jenssen, Marit R. Bjørgaas, Kathrine F. Frøslie, Kristin Godang, Håvard Kalvøy, Ørjan G. Martinsen, Hanne L. Gulseth

**Affiliations:** 1 Department of Clinical and Biomedical Engineering, Oslo University Hospital, Oslo, Norway; 2 Department of Endocrinology, Morbid Obesity and Preventive Medicine, Oslo University Hospital, Oslo, Norway; 3 Department of Organ Transplantation, Oslo University Hospital and University of Oslo, Oslo, Norway; 4 Metabolic and Renal Research Group, Faculty of Health Sciences, UiT The Arctic University of Norway, Tromsø, Norway; 5 Department of Endocrinology, St. Olavs Hospital, Trondheim University Hospital, Trondheim, Norway; 6 Department of Clinical and Molecular Medicine, NTNU – Norwegian University of Science and Technology, Trondheim, Norway; 7 Norwegian National Advisory Unit on Women’s Health, Oslo University Hospital, Oslo, Norway; 8 Department of Physics, University of Oslo, Oslo, Norway

Correction to: *Scientific Reports* 10.1038/s41598-018-33189-1, published online 03 October 2018

In Figure 2, the coloured areas representing the margins of error were omitted. In addition, in the HTML version, the black line representing the Reaction group appears blue. The correct Figure 2 appears below as Fig. 1.

**Figure 1:**
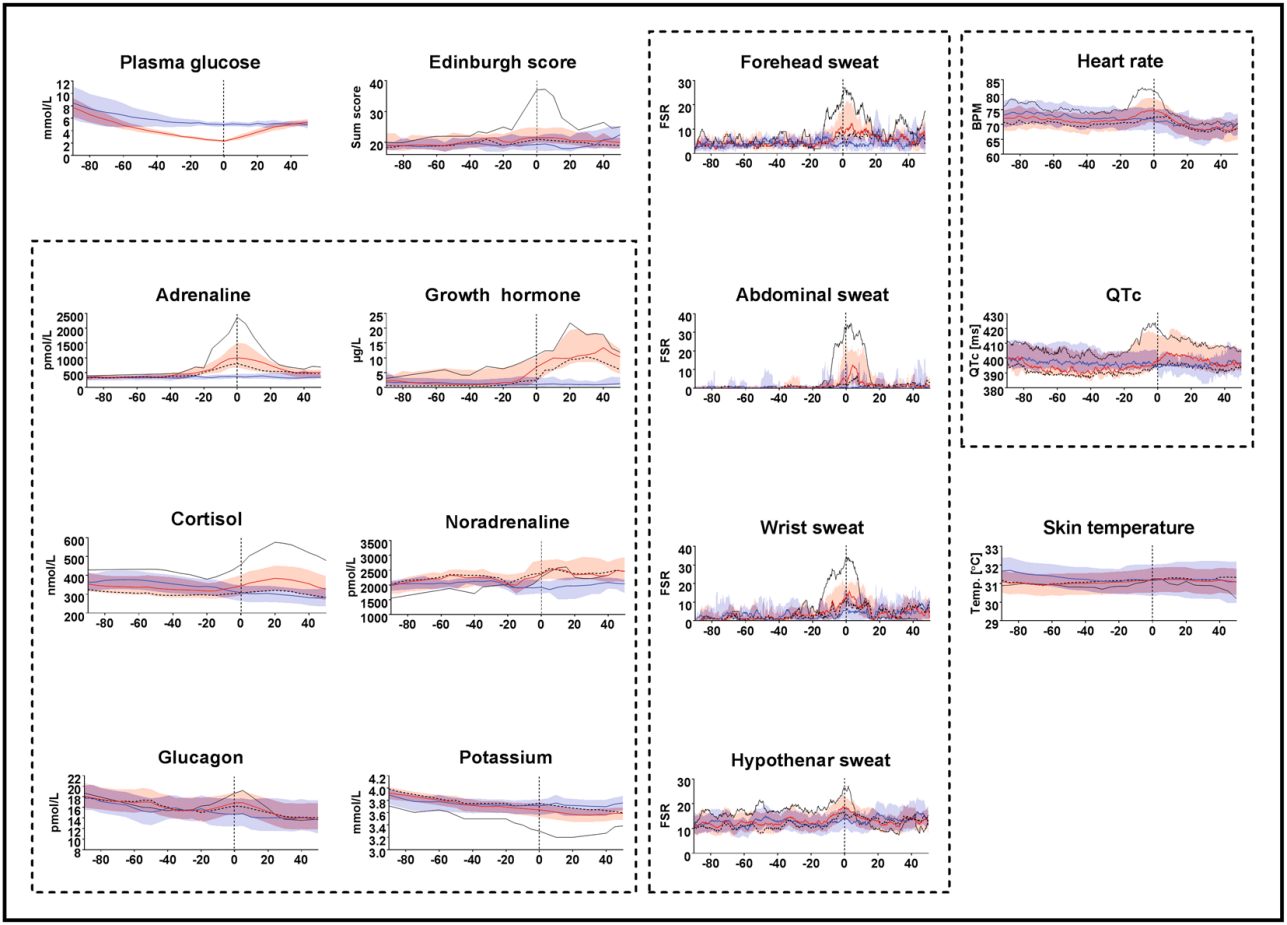
Hormonal and symptomatic responses during HYPO-clamp (red) and EU-clamp (blue). Red and blue lines: Medians (or means, if applicable). Colored areas: 95% CI. Black lines: medians or means for the Reaction group during HYPO-clamps. Dashed black lines: medians or means for the Non-reaction group during HYPO-clamps. Time = 0 is marked with a vertical dashed line. Dashed boxes circumscribe related graphs (blood tests, sweat measurements and ECG-data).

