# Supplementary material for: Evaluation of Hypoglycaemia with Non-Invasive Sensors in People with Type 1 Diabetes and Impaired Awareness of Hypoglycaemia
Source: Sci Rep. 2018 Oct 3;8:14722. doi: 10.1038/s41598-018-33189-1 (PMC6170450; doi:10.1038/s41598-018-33189-1)
Supplement: Supplementary file 1 — Supplemental material [file 41598_2018_33189_MOESM1_ESM.docx]

**Supplemental Material**.

# *Evaluation of Hypoglycaemia with Non-Invasive Sensors in People with Type 1 Diabetes and Impaired Awareness of Hypoglycaemia*

Ole Elvebakk*^1^, Christian Tronstad^1^, Kåre I. Birkeland^2^, Trond G. Jenssen^3,4^, Marit R. Bjørgaas^5,6^, Kathrine F. Frøslie^7^, Kristin Godang^2^, Håvard Kalvøy^1^, Ørjan G. Martinsen^8,1^, Hanne L. Gulseth^2^ .

^1^Department of Clinical and Biomedical Engineering, Oslo University Hospital, Oslo, Norway.

^2^Department of Endocrinology, Morbid Obesity and Preventive Medicine, Oslo University Hospital, Oslo, Norway.

^3^Department of Organ Transplantation, Oslo University Hospital, Oslo, Norway.

^4^Metabolic and Renal Research Group, Faculty of Health Sciences, UiT The Arctic University of Norway.

^5^Department of Endocrinology, St. Olavs Hospital, Trondheim University Hospital, Trondheim, Norway.

^6^Department of Clinical and Molecular Medicine, NTNU – Norwegian University of Science and Technology, Trondheim, Norway.

^7^Norwegian National Advisory Unit on Women's Health, Oslo University Hospital, Oslo, Norway.

^8^Department of Physics, University of Oslo, Oslo, Norway.

## **Fig. 1: Outline of clamp set up**
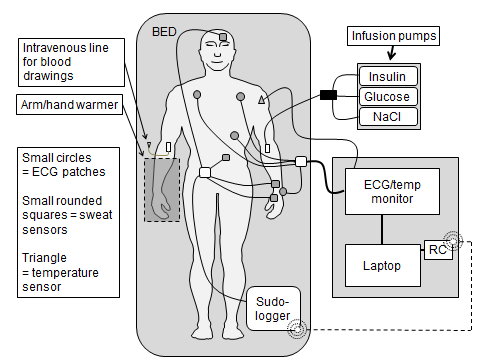


## **Note 1. List of exclusion criteria**

Participants were excluded from the study if they met any of the following criteria:

1. Below 18 or above 60 years of age.
2. Use growth hormone or thyroxine (thyroxine was allowed if the patient was euthyroid).
3. Used alfa- or beta-blockers
4. Used tricyclic antidepressants.
5. Used antihistamines, unless they could be seponated (at least a week) before the trial.
6. Used any medication containing paracetamol that cannot be seponated (at least 72 hours) before the trial.
7. Used any medication (enteral, topical (including inhalers) or injected) that contained glucocorticoids. However, short periods of for example prednisolone tablets could be tolerated, as long as it was not used for periods long enough to suppress cortisol production. But for a period of two weeks before the trial, no use of glucocorticoids in any form was allowed.
8. Have had convulsive attacks due to hypoglycaemia with blood sugar above 2.0. Epileptic seizures was not an exclusion criteria unless the last seizure was less than a year before the trial, or if for any reason the epilepsy was not considered to be under control. Anti-convulsive drugs could be accepted.
9. Suffered from Addisons disease or hypophyseal failure.
10. Women of childbearing potential (WOCBP) had to use an adequate method of contraception to avoid pregnancy throughout the study. All WOCBP had to have negative serum or urine pregnancy test at both study visits.
11. Antihypertensive medication (except alfa- and betablockers), lipid lowering drugs, oral contraceptives, hormone replacement therapy, multivitamin supplements and nutritional supplements was allowed if the participants adhered to the same regimen during the study.
12. History of substance abuse, including anabolic steroids (replacement therapy was allowed).
13. Serious mental illness.
14. History of cardiovascular disease.
15. Symptoms of heart disease, including angina, shortness of breath, fatigue, oedema, palpitations, irregular pulse, cyanosis.
16. High blood pressure (above 160 systolic or above 100 diastolic)
17. Kidney failure (GFR had to be 60 or higher).
18. Known lung disease.
19. Other chronic diseases of a serious nature.
20. Abnormal ECG.
21. Any medical condition that in the judgment of the investigator would jeopardize the participant’s safety or evaluation of the study device.

## **Note 2. Some more details on the clamp procedure.**

A total of nine blood samples for counterregulatory hormones were drawn during one clamping procedure. During hypoglycaemia, they were drawn at the beginning of the clamping procedure and then at predefined PG levels during lowering and raising of PG. A total number of nine blood samples for counterregulatory hormones were drawn during one procedure. The predefined PG levels were (during lowering of PG, in mmol/L) 4.5, 4.0, 3.5, 3.0 and 2.5 mmol/L. Fifteen minutes later another sample was drawn at 2.5 mmol/L, and then (during raising of PG) at 3.5 and 4.5 mmol/L. The last blood sample was drawn at least 30 minutes after the second to last.

In the euglycaemic clamp, blood samples were taken at the same time since the beginning of the clamp as in the hypoglycaemia clamp for the same participant. However, in half of the cases the euglycaemic clamping was done first, then the blood samples were drawn at predefined times thought to correspond in time to the ones taken in hypoglycaemic clamping. With the first sample being drawn at zero minutes (at the beginning of the clamp, in the figures time zero is set to nadir), the rest of the samples were drawn at these time points (in minutes after zero): 60, 75, 85, 100, 115, 130, 145 and 175.

## **Note 3. Determination of Sample Size.**

The power analysis is based on the expected *sweating* effect size, which is the measurement among the multi-sensor measurements which is best known in this type of experiment based on piloting and previous research. The research design is four repeated measurements, and the power analysis is based on the expected within-subject changes in sweating and the expected between-subjects variation. The power calculation is based on a repeated measurements ANOVA test, with an α=0.05 and a power (1-β)=0.9 requirement. The other input parameters (variance explained by effect, variance within group and correlation between repeated measurements) are set according to a “worst-case” situation of expected measurements. This input gives a need for 19 participants based on the following calculation in the software G*Power v3.1.6:

**F tests -** ANOVA: Repeated measures, within factors

**Analysis:** A priori: Compute required sample size

**Input:** Effect size f = 0.4082483

α err prob = 0.05

Power (1-β err prob) = 0.90

Number of groups = 1

Number of measurements = 4

Corr among rep measures = 0.2

Nonsphericity correction ε = 1

**Output:** Noncentrality parameter λ = 15.8333341

Critical F = 2.7757624

Numerator df = 3.0000000

Denominator df = 54.0000000

Total sample size = 19

Actual power = 0.9115807

Based on this analysis, 20 participants were recruited for the study.
